# Foot deformation analysis with different load-bearing conditions to enhance diabetic footwear designs

**DOI:** 10.1371/journal.pone.0264233

**Published:** 2022-03-23

**Authors:** Liying Zhang, Kit-lun Yick, Pui-ling Li, Joanne Yip, Sun-pui Ng

**Affiliations:** 1 The Institute of Textiles and Clothing, The Hong Kong Polytechnic University, Kowloon, Hong Kong, China; 2 Laboratory for Artificial Intelligence in Design, Hong Kong Science Park, New Territories, Hong Kong, China; 3 Hong Kong Community College, The Hong Kong Polytechnic University, Kowloon, Hong Kong, China; University of Illinois at Urbana-Champaign, UNITED STATES

## Abstract

In-depth analyses of foot surface measurements upon weight bearing are crucial to understand how the dorsal and plantar surfaces of the foot deform during motion to enhance the fit of footwear, which is particularly important for diabetic patients with stringent fit requirements to redistribute the plantar weight forces. This study analyzes diabetic foot deformations under 3 different weight bearing conditions (no weight bearing, half weight bearing, and 80% weight bearing) by using a novel foot scanning method that enables efficient scanning of the dorsal and plantar surfaces of the foot simultaneously. The feet of 48 patients with diabetes mellitus (DM) are scanned. With increased load on the feet, the width of the forefoot increases by 9.7%-10.4%, height of the midfoot decreases by 15.1%-18.2%, forefoot and midfoot rotate to the medial side by 16.9%-23.9% while the rearfoot rotates to the lateral side by 15.2% simultaneously, and the plantar of the foot increases contact with the floor by 11.4%-23.0%. Gender differences in foot shape are also found between males and females, males have a broader foot than females for the same foot length. Precise anthropometric information of foot changes and deformation therefore enables adequate foot protection, fit and comfort when designing footwear. This research contributes to shoe design considerations that focus on the deformation of the foot under different loads.

## Introduction

Foot ulceration is a debilitating and costly complication for diabetics. According to the International Diabetes Federation, approximately 463 million adults between 20 and 79 years old were living with diabetes in 2019 [1]. The diabetic population is anticipated to increase to 700 million in 2045 [2]. It is estimated that 15% of the population will develop a foot ulcer during their lifetime and 2–3% may develop a foot ulcer annually [2]. Diabetic foot ulcers can be caused by several factors. In addition to the poor glycemic control, other causes include calluses, improper foot care, foot deformation and ill-fitting footwear [3]. Apart from medical treatment to control the blood glucose level [4], the primary means of managing diabetic foot problem include regular foot examination and hygienic practices [5–7], patient education [8–10], prompt treatment of minor injuries [11] and appropriate footwear [12–15]. Custom-fabricated orthotic footwear with a roomy toe box has also been extensively used to prevent diabetic ulceration in clinical practices. The footwear is engineered to offer proper arch support, reduce the magnitude of pressure on diabetic wounds, and redistribute the plantar weight forces on the metatarsal heads and bony prominences which may build excessive hyperkeratotic tissues that eventually ulcerate [16–19]. Despite the many advantages and advancements in the design of diabetic footwear, foot ulcerations still occur, with a high risk of re-ulceration [20–22]. The shape of the patient’s foot, the fit of the footwear, the choice of footwear material and the adherence to footwear use are key parameters affecting the plantar offloading performance of the footwear and the prevention of recurrence of plantar injury and ulcerations in diabetic patients [23–25]. The shape of the foot, however, alters under weight-bearing and gait which not only affect the fit and comfort of footwear, but also the support and offloading effects of the footwear [26–28]. To reduce wear discomfort and fit related problems of stress during walking, extensive research has been carried out with anthropometric analyses of the foot, with foot deformation under different weight bearing conditions when standing and walking as the specific focus of study [29–32].

Due to peripheral neuropathy and vascular diseases, the structure of the foot of diabetic patients differs from that of those without diabetes [33]. Diabetic patients are prone to various foot deformations, affecting their foot anthropometric measurements and pressure parameters [34]. Sacco et al. [35] found that compared to healthy subjects, a larger proportion of diabetics have flat feet. Mueller et al. [36] examined the relationship between foot structure measurements and peak plantar pressure (PPP) during walking, and found that the metatarsal-phalangeal joint (MPJ) angle (dorsiflexion at this joint causes hammer toe deformity) is the most important variable to predict pressure of the forefoot region in the diabetes mellitus (DM) group.

These foot structural deformations also cause mismatch between foot and footwear, which can be examined by comparing the foot shape with footwear dimensions. Many diabetics wear shoes that do not fit, particularly, shoes that are too narrow for the width of their feet [37, 38]. McInnes et al. [39] and Harrison et al. [38] examined the footwear size of diabetics and found that a large proportion of them are wearing inappropriate footwear during daily activities. A smaller version of males’ footwear was traditionally designed for females [40]. However, previous studies have concluded the feet of females is not simply scaled down of males. The gender differences showed in the axis of the metatarsal heads angle and the dimensions of the arch [41].

An accurate and reliable method to obtain the foot contours is paramount for orthopedic footwear to provide optimal fit and foot protection. However, the foot shape considerably deforms with foot rotation upon weight bearing and walking. There is still much ambiguity and contradicting evidence on the reliability and accuracy of assessing foot shape and dimensions [42, 43]. Xiong et al. [32] showed that the foot length increases when no body-weight to full body-weight is placed on the foot. The foot also increases in width, reduces in height and rotates to the medial side upon loading. The foot becomes broader and flatter with increased load [32, 44]. As compared to the shape of the dorsal surface of the foot, it is anticipated that the characterization of deformations of the surface of the foot plantar during walking would be more complex and challenging. Yet scientific knowledge on the surface deformation of foot shape and measurements in dynamic situations that enable precise support and foot protection for diabetic patients is minimal.

Footwear is a cost-effective way of reducing high plantar pressure which would then prevent foot ulcers. A suitable design and fabrication of an arch support with increased contact area (CA) can therefore effectively redistribute the PPP from the forefoot to prevent neuropathic ulceration. However, there is still much ambiguity in determining the 3D geometric contours and the shape of the plantar surface with different loading conditions under various stances. In order to address this research gap, we endeavor to provide a better understanding on how the foot geometry changes with different loading conditions. This can provide a useful reference for improving the designs of diabetic footwear and insoles.

## Methods

### Participants

A total of 48 diabetes mellitus (DM) patients including 19 males and 29 females between 56 and 75 years old (mean: 65; SD: 5) participated in the study. The inclusion criteria [45, 46] are those who have Type 1 or 2 DM in the early stages (self-reported with a clinical physiotherapist diagnosis), no history of ulcers or neurological disorders (except neuropathy), and able to walk a length of 20 m continuously without a walking aid [47]. The exclusion criteria are those who have active ulcers. This study was approved by the Human Subjects Ethics Sub-committee of The Hong Kong Polytechnic University (Reference Number: HSEARS20200128001). All of the participants provided written informed consent before participation in the study.

### Experiment

**Fig 1:**
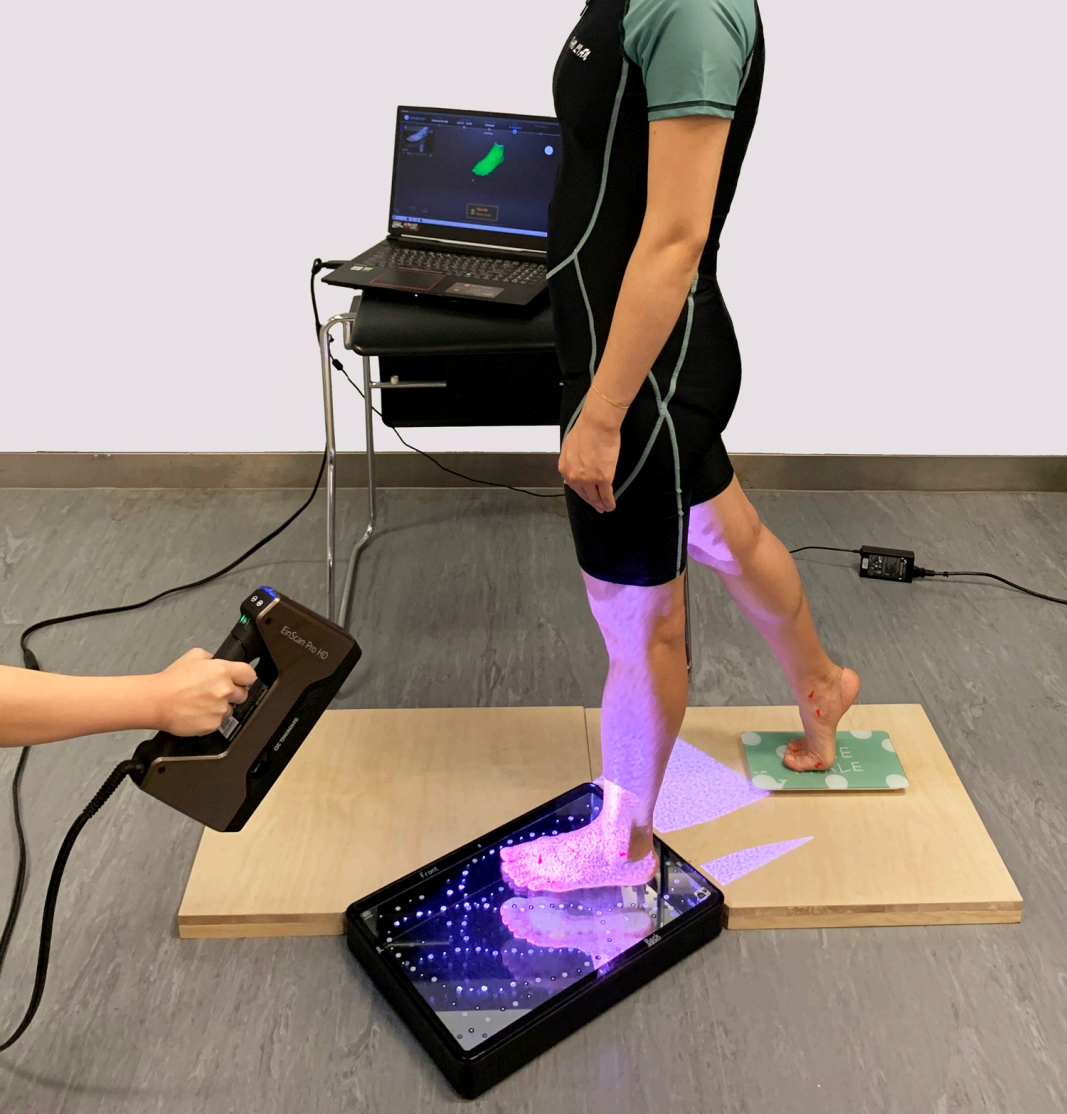
EinScan Pro HD 3D handheld scanner with foot station.

An EinScan Pro handheld (HD) 3D scanner [48, 49] with a foot station was used to obtain the foot shape. This scanner can scan a wider range of objects with new lighting projection hardware and software algorithm, including dark or black colors and casting metal surfaces. It can process up to 3,000,000 points per second under the handheld scan mode, and less than 0.5 s to capture every single frame in the fixed scan mode. By using a minimum point distance setting of 0.2 mm with the optimized algorithm, the scanner has a similar resolution and accuracy as a fixed scanner. The interface uses USB 3.0 which provides high speed data transmission by type of positioning method. Both scanner and object can be moved during scanning. The scanner delivers an accuracy of up to 0.04 mm in the fixed scan mode. Also, the novel foot station used in this study enables efficient scanning of the dorsal and plantar surface of the foot simultaneously; see Fig 1. The station consists of a mirror and a glass plane with marked points. The former facilitates visibility of the bottom of the feet and the latter helps the algorithm to locate the points for scanning. After the scanning process is completed, the algorithm will align the upper and the bottom sides of the feet together automatically.

#### Foot anatomical landmarks

**Fig 2:**
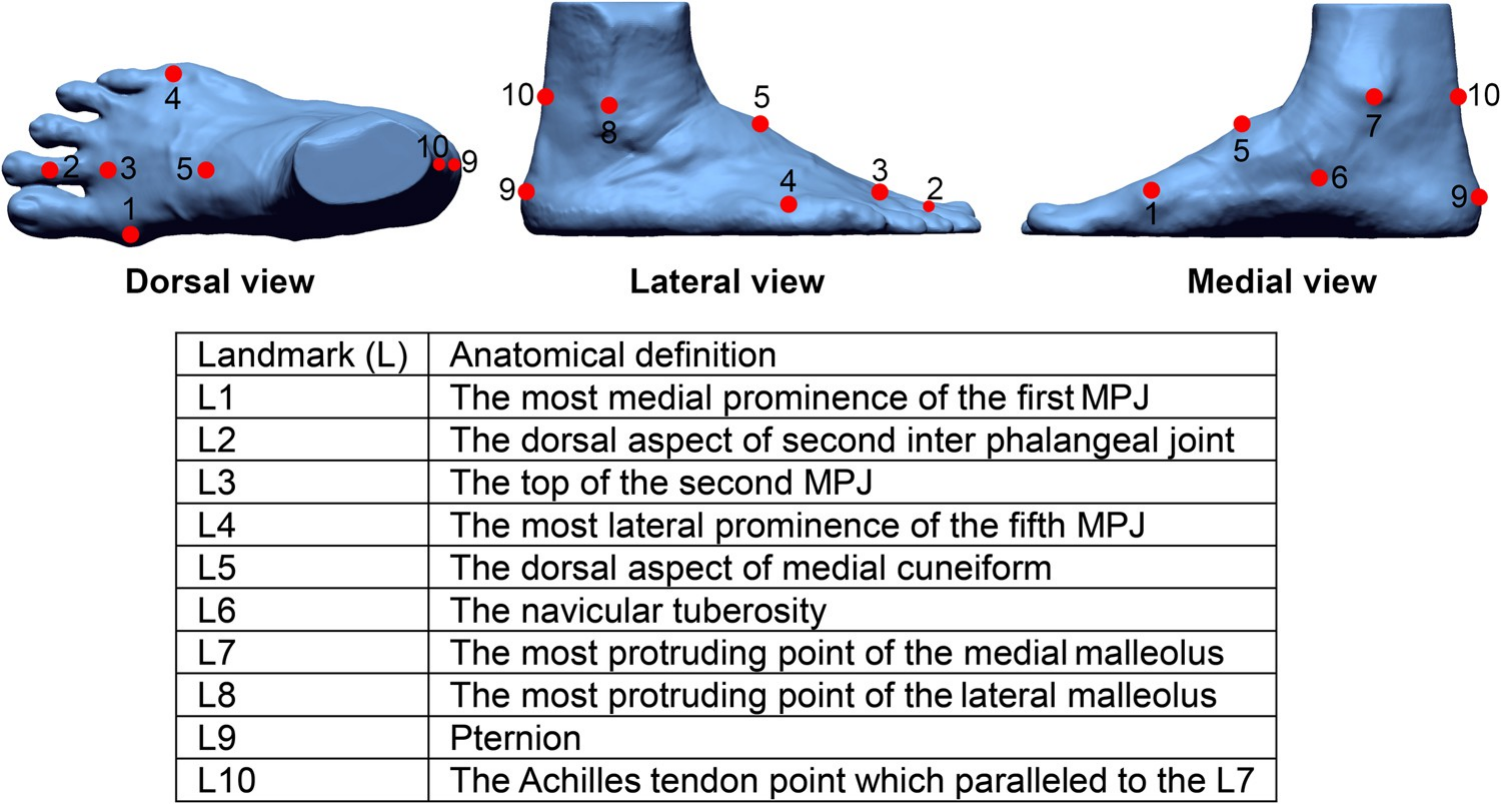
Location of 10 anatomical marker points on the right foot.

A total of ten markers were attached to each foot (Fig 2). They were placed on the most medial prominence of the first MPJ, the dorsal aspect of the second interphalangeal joint, top of the second MPJ, most lateral prominence of the fifth MPJ, dorsal aspect of the medial cuneiform, navicular tuberosity, most protruding point of the medial and lateral malleolus, pternion and Achilles tendon which is parallel to the most protruding point of the medial malleolus [32, 50].

#### Foot scanning and measurements at various weight-bearing conditions

**Fig 3:**
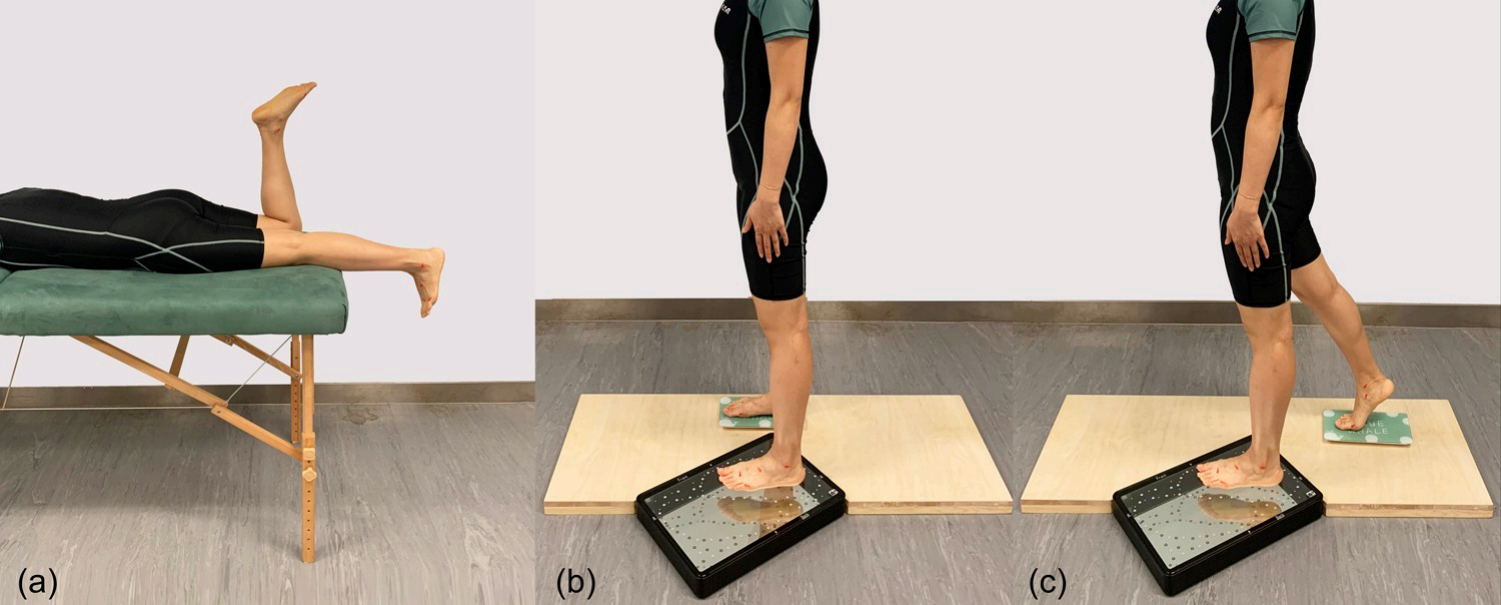
Images of three weight bearing conditions: (a) no weight bearing—NWB; (b) half weight bearing—HWB; (c) 80% weight bearing—80%WB.

All of the subjects were required to kick a ball in front of him/her to determine his/her dominant foot before the scanning process took place [32, 51]. Studies have concluded there are no significant differences between left and right foot [32, 52], moreover, the dominant foot has higher risk of foot ulceration than the non-dominant one [53, 54]. Therefore, only the dominant foot was scanned under three different weight bearing conditions (Fig 3): no weight bearing (NWB), half weight bearing (HWB), and 80% weight bearing (80%WB). NWB and HWB are commonly used in clinical casting, and the foot shape with HWB is recommended for insole designs [44]. Since full weight bearing (FWB) with single limb support in the mid stance phase is challenging to the recruited subjects, the foot was further scanned at 80%WB (at the stage of loading response). For the NWB condition, the participant laid prone on an examination bed, the foot to be scanned was in an unloaded position, and the other foot was bent perpendicular to the thigh. For the HWB condition, the subjects were requested to stand upright with equal loading on each foot [55]; For the 80% WB condition, the participants were instructed to stand with their knee flexed slightly in order to absorb the shock as the foot fell flat on the ground for stabilization in advance of single limb support. They were required to put about 80% of their weight on the dominant foot by using the weight scale [42] to control about 20% of their weight on the non-dominant foot.

**Fig 4:**
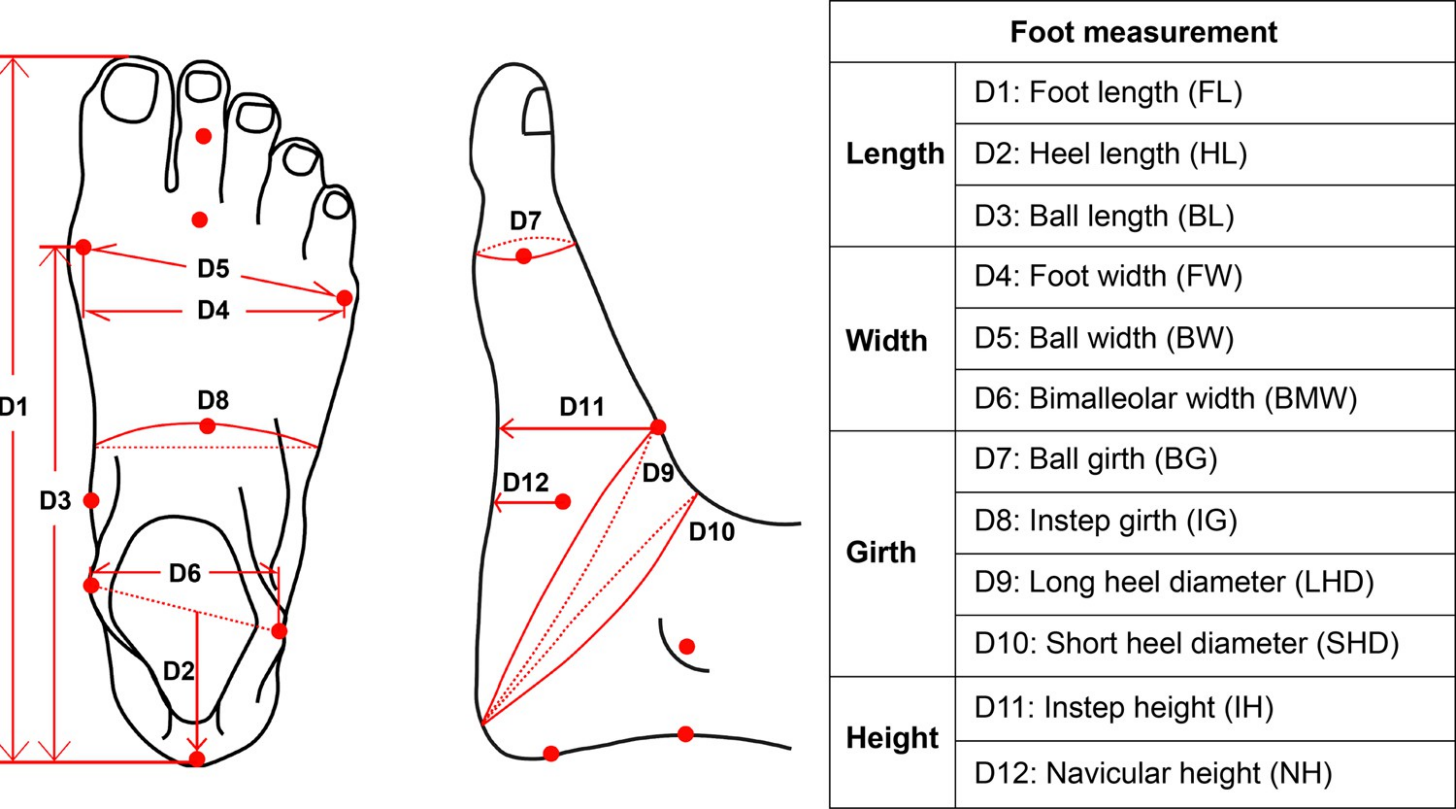
Twelve anthropometric measurements of foot taken from 3D images [59].

**Fig 5:**
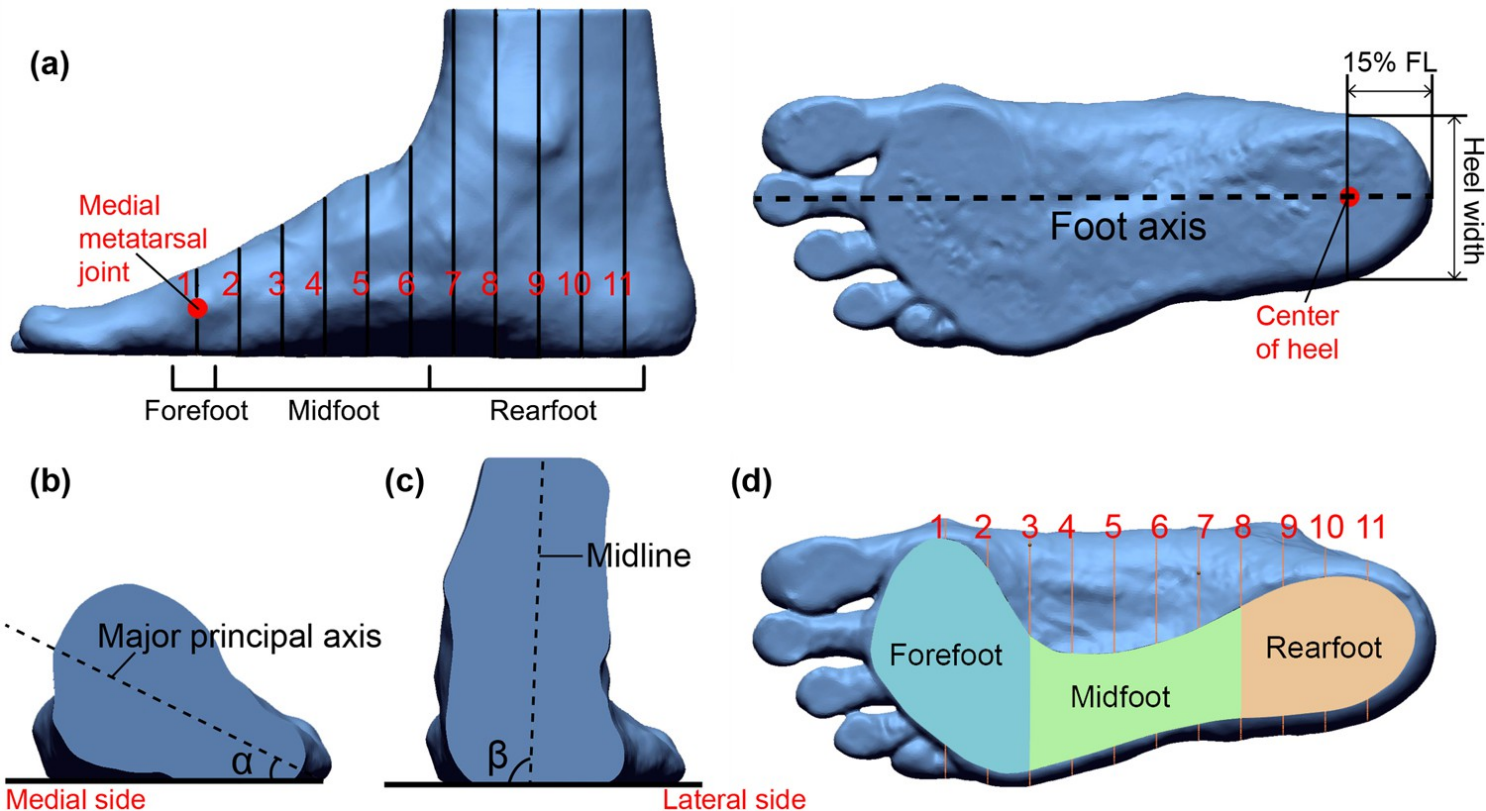
Foot segmentation and angles α and β: (a) foot is divided into 10 sections (from medial metatarsal joint to the center point of heel); (b) cross section in the midfoot and α (the angle between the major principal axis of mid foot cross section and the floor); (c) cross section in the rearfoot region and β—inclination of midline; and (d) outline and division of the contact area (plantar view).

Geomagic Design X software was used to align and measure the scanned images. All of the 3D images were aligned to a standard position [56]. Twelve foot dimensions were extracted from the 3D images under the three different weight bearing conditions (Fig 4). In addition, the rotation of the forefoot, midfoot and rearfoot under different weight bearing conditions was also evaluated. Each foot image was further split into 10 equal parts from the medial metatarsal joint to the center of the heel [57] along the foot axis (Fig 5A). The cross section that passes through the medial metatarsal joint represents the forefoot region. Cross sections 2–6 represent the midfoot region, and cross sections 7–11 represent the rearfoot region. The direction of the major principal axis of the cross sections in each region was used to determine the foot rotation. The angle between the major principal axis of each cross section and the floor was defined as α (Fig 5B). The mean α of all the cross sections in the forefoot (angle α1) and midfoot (angle α2) regions was used to represent the direction of the major principal axis [32, 58]. The angle between the midline and the floor was defined as angle β (Fig 5C), and the mean β of all the cross sections in the rearfoot region was used to represent the direction of the midline. The angles were measured through AutoCAD software. Additionally, the CA with exclusion of the toes under different weight bearing conditions are also extracted and compared (Fig 5D).

### Data analysis

IBM SPSS Statistics 21 software was used for all of the statistical analyses and the level of significance was set at 0.05. Intraclass correlation coefficients (ICCs) were used to determine the reliability of 3 repeated measurements of the same 3D image from one operator. The result showed that the ICC values measured are all higher than 0.95 (P<0.001), with good reproducibility. The normality of the 12 foot measurements, angles and contact area in each foot region under the three weight bearing conditions (NWB, HWB and 80%WB) were tested with the Shapiro-Wilk test. The results showed that all of the foot measurements are normally distributed (P > 0.05). The 12 foot dimensions were normalized to the corresponding foot length. One-way repeated measures ANOVA analysis was used to compare the mean value of the three weight bearing conditions, and determine whether there are significant (P<0.05) differences among the groups.

## Results

### Participant information

The descriptive statistics of the participants including age, body mass index (BMI), foot size and years of diagnosis are listed in Table 1. No participants were excluded on the basis of experiment criteria.

**Table 1:** Descriptive statistics of participants (n = 48).

Variable	Mean	Standard Deviation	Maximum	Minimum
**Male (N**_**1**_ **= 19)**				
**Age (years old)**	66	4	72	60
**BMI (kg/m2)**	23.1	2.4	26.8	19.3
**Foot size (EUR)**	40	1	43	38
**Years of diagnosis (DM)**	11	9	31	1
**Female (N**_**2**_ **= 29)**				
**Age (years)**	64	5	75	56
**BMI (kg/m2)**	24.0	3.9	33.4	18.2
**Foot size (EUR)**	39	1	42	37
**Years of diagnosis (DM)**	13	13	63	1

### Foot measurements under different weight bearings

Through one-way repeated measures ANOVA analysis (Table 2), compared with NWB, except for BL, the width (FW, BW and BMW) and girth (BG, IG, LHD and SHD) dimensions show significant increases, the length (HL) and height (IH and NH) dimensions show significant decreases with the HWB condition. No significant differences could be found between HWB and 80%WB. The BMW, IG and LHD of the males, normalized to the corresponding FL, are significantly larger than those of the females under each weight bearing condition. The other foot dimensions do not show a significant difference between males and females.

**Table 2:** ANOVA analysis of foot dimensions for NWB, HWB, and 80%WB groups.

Foot Dimensions (mm)	Mean (Standard Deviation)			HWB vs NWB (%)	80%WB vs NWB (%)	80%WB vs HWB (%)
	NWB	HWB	80%WB			
**Male (N**_**1**_ **= 19)**						
**FL**	243.2 (8.8)	246.1 (8.2)	246.2 (8.2)	1.2%	1.2%	0.0%
**HL**	72.7 (4.2)	71.9 (4.1)	71.9 (4.3)	**-1.2%**	**-1.1%**	0.1%
**BL**	179.5 (7.1)	182.6 (6.9)	182.6 (6.9)	1.7%	1.7%	0.0%
**FW**	85.7 (5.9)	93.8 (5.9)	93.9 (5.9)	**9.4%**	**9.6%**	0.2%
**BW**	88.2 (5.7)	96.9 (5.8)	97.1 (5.8)	**9.9%**	**10.1%**	0.2%
**BMW**	67.7 (3.8)	70.1 (3.8)	70.2 (3.8)	**3.5%**	**3.7%**	0.2%
**BG**	233.5 (11.9)	241.9 (12.0)	242.0 (12.0)	**3.6%**	**3.7%**	0.0%
**IG**	237.7 (10.8)	244.4 (11.0)	244.4 (11.0)	**2.8%**	**2.8%**	0.0%
**LHD**	341.8 (13.8)	351.9 (14.6)	351.9 (14.6)	**3.0%**	**3.0%**	0.0%
**SHD**	315.1 (14.0)	323.6 (14.5)	323.6 (17.2)	**2.7%**	**2.7%**	0.0%
**IH**	68.0 (5.0)	58.5 (5.1)	58.5 (5.1)	**-13.9%**	**-13.9%**	0.0%
**NH**	49.2 (8.2)	41.9 (6.8)	41.9 (6.8)	**-14.7%**	**-14.8%**	0.0%
**Female (N**_**2**_ **= 29)**						
**FL**	231.3 (9.7)	234.3 (10.1)	234.4 (10.1)	1.3%	1.3%	0.0%
**HL**	67.7 (5.3)	66.7 (5.2)	66.7 (5.1)	**-1.4%**	**-1.5%**	-0.1%
**BL**	168.8 (7.9)	171.8 (8.0)	171.8 (8.0)	1.8%	1.8%	0.0%
**FW**	82.7 (5.2)	90.7 (6.1)	90.9 (6.1)	**9.7%**	**9.8%**	0.2%
**BW**	84.7 (5.0)	93.5 (6.2)	93.6 (6.2)	**10.4%**	**10.6%**	0.2%
**BMW**	62.7 (3.6)	65.5 (3.8)	65.6 (3.8)	**4.3%**	**4.6%**	0.2%
**BG**	221.4 (10.6)	231.5 (12.2)	231.6 (12.2)	**4.6%**	**4.6%**	0.0%
**IG**	222.5 (11.5)	228.8 (12.1)	228.9 (12.1)	**2.8%**	**2.9%**	0.0%
**LHD**	320.0 (13.3)	329.2 (14.4)	329.3 (14.4)	**2.9%**	**2.9%**	0.0%
**SHD**	297.3 (13.7)	306.6 (14.6)	306.6 (14.6)	**3.1%**	**3.2%**	0.0%
**IH**	64.1 (4.3)	53.9 (3.6)	53.9 (3.6)	**-15.9%**	**-16.0%**	0.0%
**NH**	47.6 (6.1)	37.9 (5.4)	37.8 (5.4)	**-20.5%**	**-20.5%**	-0.1%

^a^ Groups with significant differences at the 0.05 level are bolded.

**Fig 6:**
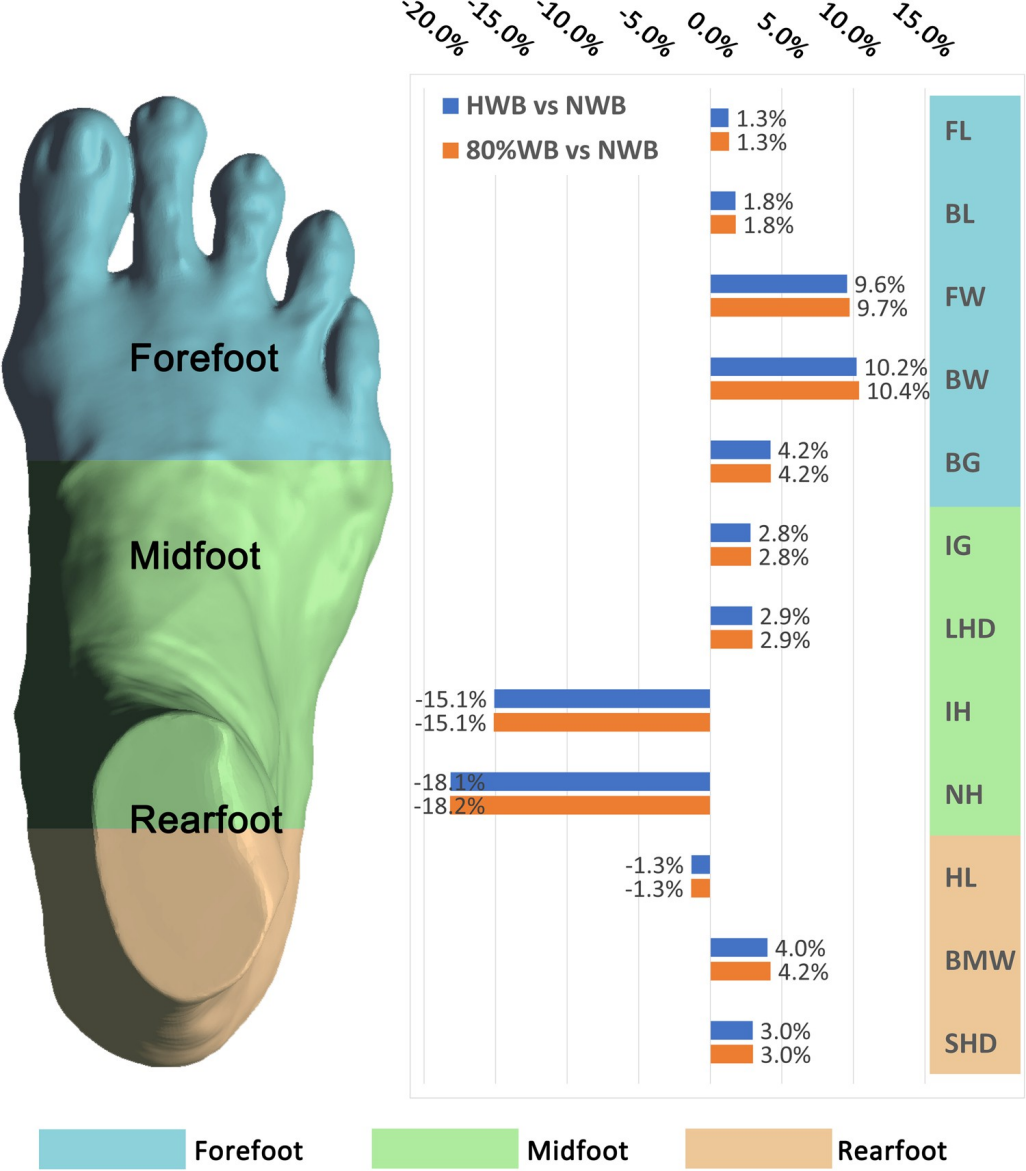
Percentage change of foot measurements.

Fig 6 shows the change percentage of all the foot measurements from NWB to 80%WB. With the 80%WB condition, for the length dimensions, the FL and BL increase by 3.1 mm (1.3%) and 3.1 mm (1.8%) respectively, the HL is reduced by 0.9 mm (1.3%) compared to the NWB. For the width dimensions, compared to the NWB, the FW, BW and BMW increase by 8.2 mm (9.7%), 8.9 mm (10.4%) and 2.7 mm (4.2%) respectively. For the girth dimensions, compared to the NWB, the BG, IG, LHD and SHD increase by 9.6 mm (4.2%), 6.5 mm (2.8%), 9.6 mm (2.9%) and 9.1 mm (3.0%) respectively. For the height dimensions, the IH and NH decrease by 9.9 mm (15.1%) and 8.8 mm (18.2%) respectively compared to the NWB. Compared with NWB, the changes in foot measurements obtained from HWB and 80%WB conditions are consistent, however, the change percentage between the two conditions is less than that between NWB and 80%WB.

#### Foot rotation under different weight bearings

**Fig 7:**
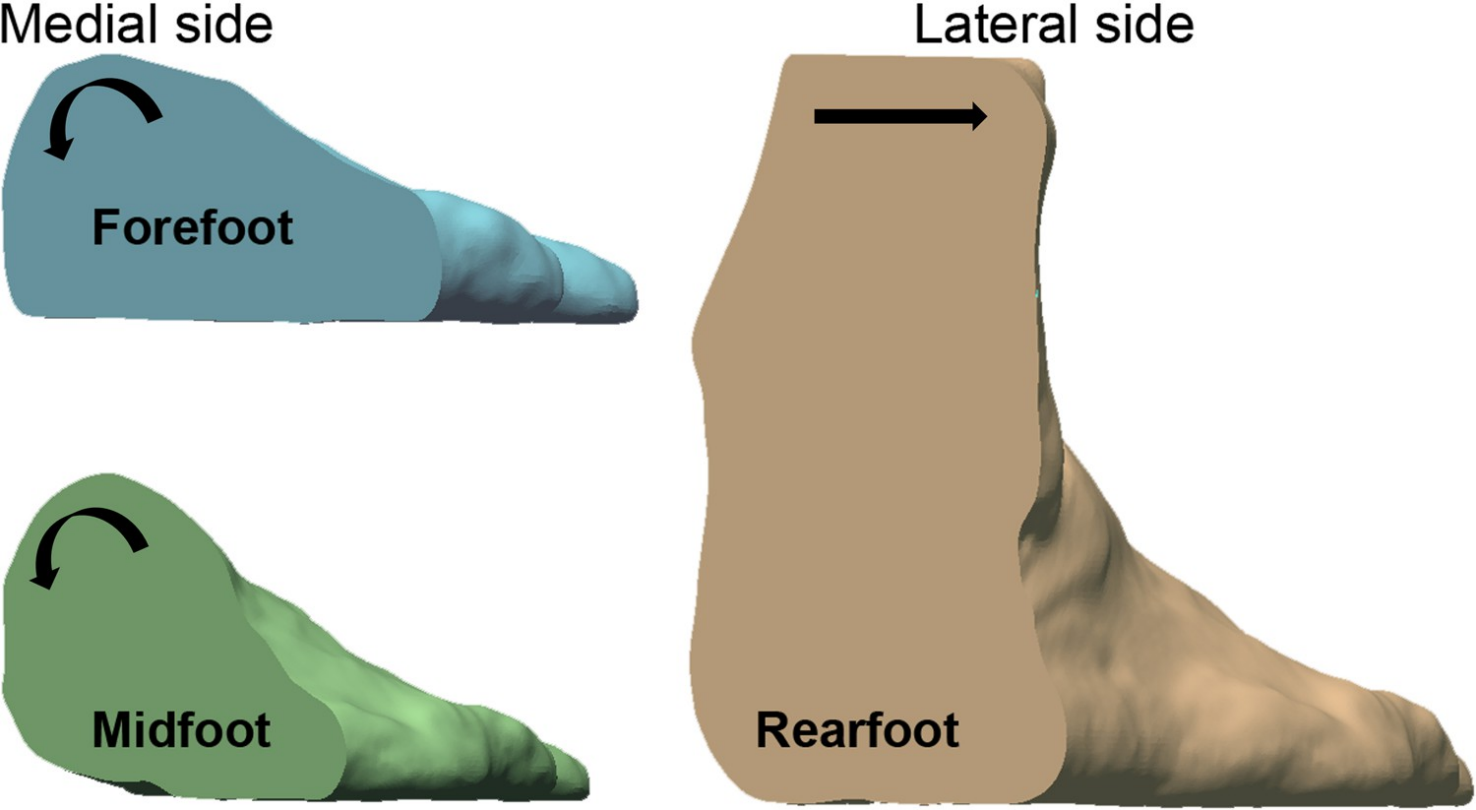
Rotation of the right foot in a cross section of the foot from NWB to HWB and 80%WB.

Table 3 show the descriptive statistics (mean, standard deviation) and change percentage of the mean angle value of each foot region. Based on one-way repeated measures ANOVA analysis, no significant differences are found between the males and females. There are significant differences with increased load on the foot in each foot region. The foot of the males and females has a similar rotation from NWB to 80%WB. In the forefoot region, the mean angle α_1_ is reduced by 2.1° (23.9%); the mean angle α_2_ of the midfoot region is reduced by 4.0° (16.9%); and the mean angle β of the rearfoot is increased by 11.9° (15.2%). The results indicate that the forefoot and midfoot rotate to the medial side with load increases, and the rearfoot simultaneously rotates towards the lateral side (Fig 7).

**Table 3:** ANOVA analysis and change percentage of angle for NWB, HWB, and 80%WB groups.

Angle (°)	Mean (Standard Deviation)			HWB vs NWB (%)	80%WB vs NWB (%)	80%WB vs HWB (%)
	NWB	HWB	80%WB			
**Male (N**_**1**_ **= 19)**						
**α1**	8.8 (1.4)	7.5 (1.5)	6.7 (1.5)	**-15.0%**	**-24.0%**	**-10.6%**
**α2**	23.4 (1.4)	20.8 (1.3)	19.7 (1.4)	**-10.9%**	**-15.8%**	**-5.5%**
**β**	78.4 (2.9)	86.4 (2.2)	90.4 (2.6)	**10.3%**	**15.4%**	**4.6%**
**Female (N**_**2**_ **= 29)**						
**α1**	8.9 (1.5)	7.5 (1.4)	6.8 (1.3)	**-15.8%**	**-23.9%**	**-9.6%**
**α2**	23.5 (1.4)	20.6 (1.3)	19.4 (1.3)	**-12.4%**	**-17.7%**	**-6.1%**
**β**	78.4 (4.5)	85.7 (2.2)	90.2 (2.7)	**9.3%**	**15.0%**	**5.3%**

^a^ Groups with significant differences at the 0.05 level are bolded.

### Plantar contact area under different weight bearings

**Fig 8:**
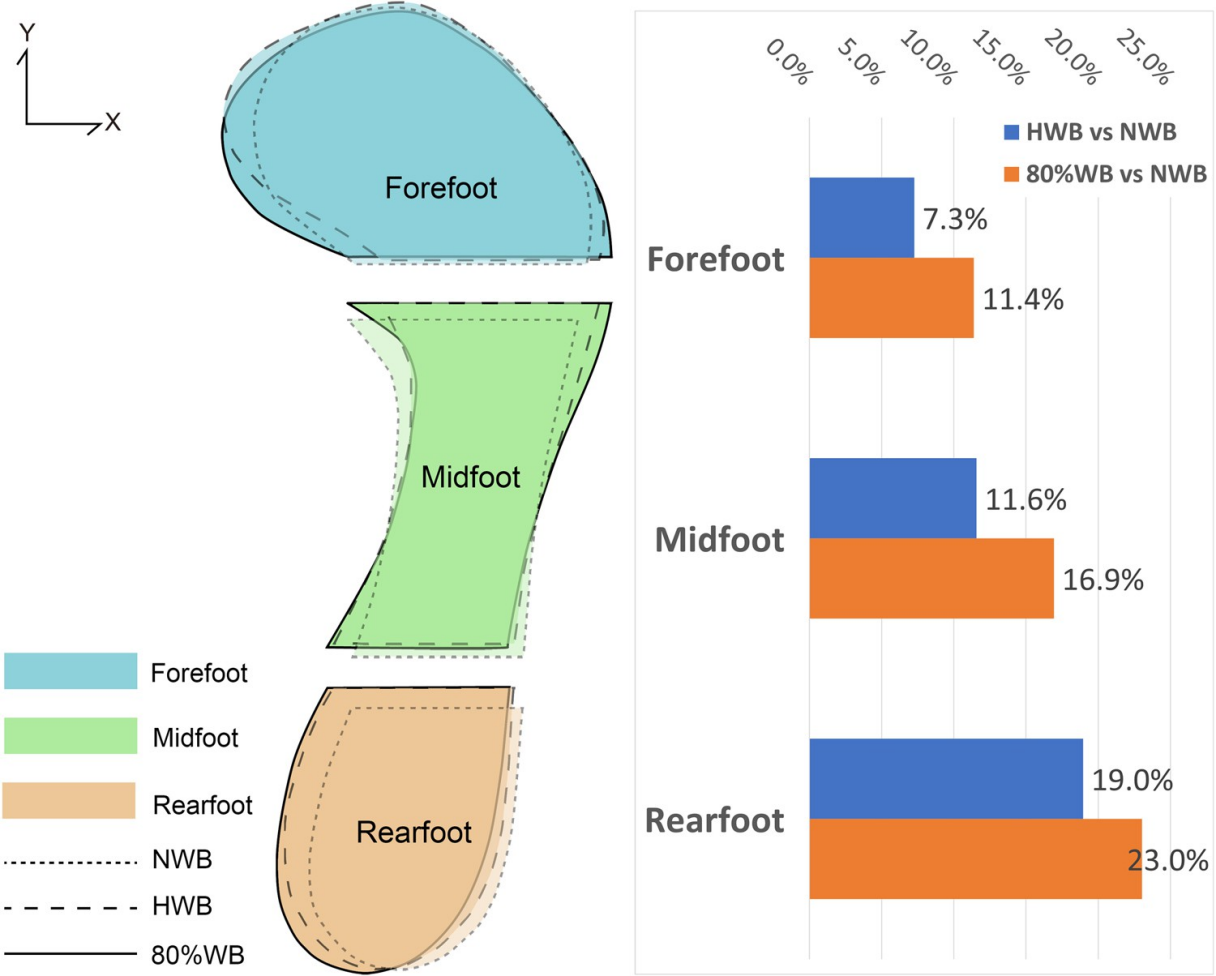
Deformation of the plantar surface of the foot (contact area).

Table 4 lists the descriptive statistics (mean, standard deviation) and change percentage of the contact area in each foot region. The result of one-way repeated measures ANOVA analysis showed significant gender specific differences. The contact area in each region of the males is larger than that of the females. However, the two groups show similar trends. The contact area in each foot region shows a significant increase from NWB to 80%WB. The mean contact area of the two groups increases by 364.7 mm^2^ (11.4%), 482.8 mm^2^ (16.9%) and 501.4 mm^2^ (23.0%) for the forefoot, midfoot and rearfoot regions respectively. The results show that the plantar of the feet has increased contact with the floor with increased load on the feet, see Fig 8.

**Table 4:** ANOVA analysis and change percentage of contact area for NWB, HWB, and 80%WB groups.

Contact area (CA) Unit: mm2	Mean (Standard Deviation)			HWB vs NWB (%)	80%WB vs NWB (%)	80%WB vs HWB (%)
	NWB	HWB	80%WB			
**Male (N**_**1**_ **= 19)**						
**Forefoot**	3359.5 (342.7)	3573.0 (363.6)	3687.6 (352.8)	**6.4%**	**9.8%**	**3.2%**
**Midfoot**	3098.3 (467.4)	3391.8 (543.6)	3514.9 (533.6)	**9.5%**	**13.4%**	**3.6%**
**Rearfoot**	2388.5 (295.1)	2748.5 (267.6)	2848.5 (256.6)	**15.1%**	**19.3%**	**3.6%**
**Female (N**_**2**_ **= 29)**						
**Forefoot**	3100.1 (417.0)	3344.8 (386.1)	3488.8 (427.1)	**7.9%**	**12.5%**	**4.3%**
**Midfoot**	2683.6 (529.4)	3038.6 (745.5)	3211.2 (817.0)	**13.2%**	**19.7%**	**5.7%**
**Rearfoot**	2037.1 (238.6)	2484.0 (301.2)	2565.7 (334.0)	**21.9%**	**25.9%**	**3.3%**

^a^ Groups with significant differences at the 0.05 level are bolded.

## Discussion

Orthotic footwear and insoles for diabetic patients are engineered designed to protect the foot from injuries with optimal fit and comfort for both static and dynamic activities. Nevertheless, the foot deforms and rotates with different weight bearing conditions [32, 42, 44]. Houston et al. [42] found that in a group of 40 young male subjects, their principal foot dimensions change significantly in the initial phases of loading (0%-10%), while the changes are less than or equal to the initial changes that take place in the later phases of loading (25%-100%). Xiong et al. [32] and Tsung [44] indicated that compared to the NWB, the foot shape with HWB/FWB changes significantly. Hence, insole design with reference to the foot shape obtained from HWB is recommended. Nevertheless, the influence of loading conditions in relation to the 3D geometric contours and the shape of the plantar foot of diabetic patients has not been fully investigated. This study uses a 3D scanning technique to simultaneously investigate the changes to the dorsal and plantar surface of the foot. Foot anthropometric measurements, rotation angle and contact areas in the NWB, HWB and 80%WB conditions are systematically compared and analyzed. It is anticipated that the 3D geometry of the foot surface and its corresponding changes with different loading conditions can provide valuable information to improve the design and fit of footwear in walking, thus allowing diabetic patients to maintain consistent plantar pressure values and reducing the risk of foot ulcerations.

### Changes in foot shape under different weight bearing conditions

In this study, statistically significant differences are found in the diabetic foot dimensions with loads compared with the NWB except for BL. When applying load on the foot from NWB to 80% WB, the forefoot and midfoot regions show a larger change percentage than the rearfoot. The width of the forefoot is increased and height of the midfoot is decreased. The forefoot and the midfoot rotate to the medial side, with the rearfoot rotating to the lateral side. In addition, the plantar of the foot shows increased contact with the floor. These results are consistent with previous studies [32, 44]. There are no significant differences between the HWB and 80%WB and the deformation between the NWB and HWB is larger than that between the HWB and 80%WB, which indicates that the foot shape with HWB can be used as a reference for footwear/insole design and selection, which is in agreement with Tsung [44].

Deformation with increased load account for foot skeleton changes. This can be explained by using the twisted plate concept, which was proposed by Michael Aloysius [60]. This arrangement of the bones of the feet allows the edge of the metatarsal heads to come into contact with the floor horizontally and the calcaneus edge placed vertically to the floor. The foot oriented as a twisted plate spreads out when bearing weight [60]. Individuals with diabetes experience extension deformity of the 1st, 2nd, and 3rd metatarsophalangeal joints [61]. The loading can flatten the medial longitudinal and transverse arches [62], then resulting in a decrease in the height with a related increase in length, while widening the foot. The dimensions changed slow down with further increase in load due to limited joint space and mobility of the foot ligaments. Ito et al. [63] quantified the changes in the foot bones under a static weight-bearing condition. All of the bones shift downward with increased load on the foot, but the vertical shift of the tibia, talus and navicular is larger than that of the calcaneus and cuboid. The talus receives the loads from tibia and fibula and passes the loads to the calcaneus and navicular [64]. The talonavicular joint tends to be about double the load passing through the calcaneocuboid joint due to the talus is more to the navicular side [62]. So the medial side of the foot is more flattened than the lateral side with the loads. The foot showed a rotation towards the medial side. Moreover, the flattened arches also led to an increase in the contact area between the plantar of the foot and the floor.

Considering the foot dimensional changes under different loads, the corresponding tolerance should be based on the material when designing footwear/insoles. Materials with higher stretchability are recommended for areas with large dimensional changes, especially the forefoot. Footwear fasteners such as shoelaces, straps or hooks-and-loops closure can also be used for fit adjustments [13]. Besides dimensional changes, spatial changes with increased load should also be considered. Foot rotation with increased load may lead to an increase in pressure on the medial side of the forefoot and the lateral side of the rearfoot. Therefore, consideration should be given to design a shoe structure to accommodate the rotation for optimal fit and support as the use of rigid footwear and insole materials may exert high levels of pressure onto the soft tissues of the foot. Tsai et al. [65] suggested that using different insole materials in different foot regions can better redistribute the foot pressure. Features with offloading effects like medial arch support and metatarsal bars can be applied in the lower pressure regions [66–68]. Soft materials with cushioning effects can be used in high pressure regions [69]. In addition, the increase in contact area between the plantar of the foot and the floor with increased load should be considered. Studies have concluded that contoured insoles are significantly better than flat insoles in reducing the local peak pressure. Studies have also shown that insoles with an arch profile could increase the contact area to reduce the plantar pressure [44, 70, 71].

### Gender specific foot shape changes

Males and females have different foot and ankle structures [72]. As such, significant differences are found in the BMW, IG, LHD and contact area between the males and females in this study. The normalized BMW, IG, and LHD values of the males are larger than those of the females. The males also have larger IG, LHD and BMW values than the females with the same FL. The result is consistent with that in Wunderlich and Cavanagh [41], in which males have a larger bimalleolar breadth and instep circumference with the same FL. These results indicate that the foot shape of males and females are not simply scaled for each dimension [41, 73]. Males tend to weigh more so their contact area is significantly larger than their female counterparts under each weight bearing condition. These results suggest that shoe manufacturers should consider gender differences to design appropriate shoes.

Nevertheless, there are limitations of this research work. First, the sample size of DM patients in this study is comparably small and they are older people who range between 60 and 75 years old. The internal and external rotations of the feet of the participants are not evaluated and classified in advance. In addition, foot deformation is reported under different loads in static conditions, which may differ during dynamic activities. Therefore, this might have limited the generalizability of the results. Further studies on the foot deformation of diabetics during dynamic activities (walking/running etc.) are recommended. This study uses a 3D scanning technique with a foot station to investigate the 80%WB conditions by using a weight scale, which allows participants to hold their foot position and keep it unflinchingly still for 60 seconds during the scanning process. However, the tenseness of the foot and the toes with little variation in position and weight bearing could also affect the result. Further studies that involve a larger number of subjects by using a dynamic foot scanning system are recommended. Although the scope of this evaluation protocol is limited, this study provides preliminary evidence that addresses the influence of different weight bearing conditions under various stances on foot dimensions and shape geometry, thereby providing the basis to advance the engineered design of footwear and insoles for optimum fit and comfort.

## Conclusion

In the static condition, the foot dimensions have different degrees of deformation with increased loading on the foot, especially the width of the forefoot and the height of the midfoot. The shape of the foot when subjected to the HWB condition have reference significance for footwear/insole design and size selection. Manufacturers should fully exploit the deformation results to select material and design proper orthopedic devices to provide a better fit, and prevent the negative effects caused by different types of deformation. For example, to accommodate a wider forefoot and collapsed arch, a broader toe box and insole with arch support are recommended. For foot rotation with increased load, a soft metatarsal pad, orthopedic insert and vamp materials with better stretching properties can prevent improper compression to the soft tissues. In addition, the foot shape is gender specific, so gender should also be considered in designing diabetic footwear.

## Supporting information

S1 File (DOCX)

S1 Data (XLSX)
