# Supplementary material for: Foot deformation analysis with different load-bearing conditions to enhance diabetic footwear designs
Source: PLoS One. 2022 Mar 23;17(3):e0264233. doi: 10.1371/journal.pone.0264233 (PMC8942268; doi:10.1371/journal.pone.0264233)
Supplement: S1 File — (DOCX) [file pone.0264233.s001.docx]

**Laboratory Protocol**

**Inclusion Criteria**

**Participants can be included in this study if they:**

- have Type 1 or 2 DM in the early stages (self-reported with a clinical physiotherapist diagnosis)

AND

- no history of ulcers or neurological disorders (except neuropathy)

AND

- able to walk a length of 20 m continuously without a walking aid

**Anonymous participant IDs**

Each participant should be given an anonymous ID according to the following naming structure: *MELD_ [site code] _number*

*[site code] = W for laboratory at The Hong Kong Polytechnic University*

*[number] = 001, 002 etc.*

Examples of participant IDs:

*MELD_W_001*

*MELD_W_002*

**Exclusion Criteria**

**Participants cannot be included in this study if:**

- have active ulcers

**Procedures**

1. *Participants were interviewed to obtain their demographic information, including name, age, BMI (body mass index), foot size and years of diabetes mellitus diagnosis*
2. *A total of ten markers were attached to each foot to ensure a reliable foot measurement*
3. *Record the body weight of participants and require him/her to kick a ball in front of him/her to determine their dominant foot*
4. *The dominant foot under three weight bearing conditions (no weight bearing, half weight bearing and 80% weight bearing) will be scanned.*
5. *At the no weight bearing condition, the participant laid prone on an examination bed, the foot to be scanned was in an unloaded position, and the other foot was bent perpendicular to the thigh.*
6. *At the half weight bearing condition, the subjects were requested to stand upright with equal loading on each foot.*
7. *At the 80% weight bearing condition, the participants were instructed to stand with their knee flexed slightly to absorb the shock as the foot fell flat on the ground for stabilization in advance of single limb support. They were required to put about 80% of their weight on the dominant foot by using the weight scale to control about 20% of their weight on the non-dominant foot.*
